# Association between Dietary Zinc Intake and Hyperuricemia among Adults in the United States

**DOI:** 10.3390/nu10050568

**Published:** 2018-05-05

**Authors:** Yiying Zhang, Yan Liu, Hongbin Qiu

**Affiliations:** 1 School of Public Health, Jiamusi University, Jiamusi 154007, China; yiyingzhang@hrbmu.edu.cn; 2 School of Public Health, Harbin Medical University, Harbin 150081, China; liuyan@ems.hrbmu.edu.cn

**Keywords:** hyperuricemia, zinc, NHANES, cross-sectional study

## Abstract

We aim to explore the associations between dietary zinc intake and hyperuricemia (HU) in United States (US) adults. 24,975 US adults aged 20 years or older from the National Health and Nutrition Examination Survey (NHANES) from 2001 to 2014 were stratified into quintiles based on zinc intake. All dietary intake measured through 24-h dietary recalls. Multivariable logistic regression analysis was performed to examine the association between zinc intake and HU after adjustment for possible confounders. For males, compared with respondents consuming less than 7.33 mg zinc daily, the adjusted odds ratios (ORs) were 0.83 (95% CI, 0.71, 0.97) among those consuming 10.26–13.54 mg zinc daily, 0.78 (95% CI, 0.63–0.96) among those consuming 18.50 mg or greater, and *p* for the trend was 0.0134. For females, compared with respondents consuming less than 5.38 mg zinc daily, the OR was 0.78 (95% CI, 0.63, 0.97) among those consuming 9.64–12.93 mg zinc daily, and *p* for the trend was 0.3024. Our findings indicated that dietary zinc intake is inversely associated with HU in US men and women, independent of some major confounding factors.

## 1. Introduction

Uric acid is the final product of purine metabolism, hyperuricemia (HU) happens while the level of serum uric acid beyond the normal range. HU is regarded as a precursor of gout and a risk factor for several chronic diseases such as chronic kidney disease, hypertension, metabolic syndrome, cardiovascular disease, and type 2 diabetes [1,2,3,4,5,6]. Epidemiological research has shown that the prevalence of HU was above 21% in American adults [7], and ranged from 13% to 25.8% in some Asian countries [8,9,10,11]. HU is becoming a significant health problem and is getting more attention. However, the pathogenesis of HU has not yet been wholly elucidated.

Zinc is an essential micronutrient that is involved in regulating inflammatory cytokines, controlling oxidative stress, and regulating immune responses [12,13,14]. Zinc deficiency is involved in growth retardation, cell-mediated immune dysfunction, and cognitive impairment [15]. Uric acid as a major antioxidant in the human plasma or pro-oxidant within the cell [16], may be associated with zinc, which has the potential to retard the oxidative process [17]. A growing body of evidence indicates that serum zinc may be associated with the level of serum uric acid. A report revealed that oral zinc therapy can produce an improvement in hypouricemia in patients with Wilson’s disease by increasing uric acid synthesis in the liver [18]. An animal study reported that the serum uric acid levels of diabetic rats treated with zinc-flavonol complex were reverted back to near normalcy [19]. A longitudinal research demonstrated that uric acid was negatively linearly related to serum zinc in hemodialysis patients [20]. However, studies investigating the relationship between dietary zinc intake and risk of HU are scarce, only one cross-sectional study has shown an inverse association between dietary zinc intake and HU in middle-aged and older men in China [21].

No known studies have examined the relationships between dietary zinc intake and HU for the American population. Therefore, the aim of this cross-sectional study was to investigate this relationship in a large population-based US study with a hypothesis that dietary zinc intake is inversely associated with HU.

## 2. Materials and Methods

### 2.1. Study Populations

Data from the National Health and Nutrition Examination Survey (NHANES), which is an ongoing, continuous survey with data released in two-year cycles. NHANES is a cross-sectional series of interviews and examinations of the non-institutionalized civilian population in the United States (US), managing by the Centers for Disease Control and Prevention (CDC) [22]. NHANES is a publicly available dataset. The data for these surveys including interviews, physical and laboratory examination can be downloaded from the NHANES website (http://www.cdc.gov/nchs/nhanes.htm). Data accumulation was performed by the National Center for Health Statistics with approval from their ethics review board [23], and additional Institutional Review Board approval for the secondary analyses was not required [24].

A total of 37,215 adults aged 20 years or older provided uric acid samples for NHANES from 2001 to 2014. Excluded were pregnant women (*n* = 1507); participants with a missing or incomplete essential information on demographic or total nutrient intakes dietary interview (*n* = 8912); those taking medications that might affect uric acid metabolism, such as furosemide, losartan, and allopurinol (*n* = 1420); and those with serum creatinine >1.5 mg/dL [25] were also excluded for considering renal dysfunction (*n* = 401). After exclusion, the total subjects in our study included 24,975 adults (12,218 women, 12,757 men).

### 2.2. Study Variables

According to the zinc intake quintiles, the patients were divided into five groups: ≤7.33 mg, 7.34–10.25 mg, 10.26–13.54 mg, 13.55–18.49 mg, and ≥18.50 mg daily in males; and ≤5.38 mg, 5.39−7.37 mg, 7.38–9.63 mg, 9.64–12.93 mg, and ≥12.94 mg daily in females. Participants without hypertension or diabetes were also divided into five groups: ≤7.67 mg, 7.68–10.66 mg, 10.67–13.98 mg, 13.99–19.08 mg, and ≥19.09 mg daily in males; ≤5.54 mg, 5.55–7.54 mg, 7.55–9.86 mg, 9.87–13.17 mg, and ≥13.18 mg daily in females; ≤6.60 mg, 6.61–9.46 mg, 9.47–12.42 mg, 12.43–16.99 mg, and ≥17.00 mg daily in males with hypertension or diabetes; and ≤5.01 mg, 5.02–6.98 mg, 6.99–9.03 mg, 9.04–12.20 mg, and ≥12.21 mg daily in females with hypertension or diabetes. Recommended dietary allowance (RDA) of zinc intake was developed by the Food and Nutrition Board (FNB) of the Institute of Medicine and was varied by gender and age. For US adults, RDAs for zinc were 11 mg/day for male and 8 mg/day for female aged 19 years and above [26]. We also divided participants into two groups according to RDAs of zinc: <11 mg/day and ≥11 mg/day in male; <8 mg/day and ≥8 mg/day in female. All patients were interviewed by the first 24-h dietary recall to obtain total nutrient intakes through in-person interviews from 2001 to 2014, and a part of the adult participants participated in second dietary surveys through the telephone interviews 3 to 10 days after the initial recall interview since 2003. We used the first 24-h dietary recall to obtain total nutrient intakes, including their intake of zinc, energy, protein, carbohydrate, vitamin C, vitamin E, and dietary fiber. Because the first 24 h dietary recall may cause a bias against the estimation of nutrient intake, we undertook sensitivity analyses among the respondents whom provided a second dietary recall. Physical examinations such as weight, height, and blood pressure were conducted following standardized protocol. Body mass index (BMI) was defined as weight divided by height^2^ (kg/m^2^). Hypertension was defined as systolic blood pressure ≥140 mm Hg or diastolic blood pressure ≥90 mm Hg. HU was defined as serum uric acid ≥7.0 mg/dL in males and ≥6.0 mg/dL in females. Serum concentrations of uric acid were detected on a Beckman UniCel^®^ DxC800 Synchron or a Beckman Synchron LX20 (Beckman Coulter, Inc., Brea, CA, USA) after oxidation of uric acid by uricase to form allantoin and H_2_O_2_. The covariates included age, race/ethnicity (categorized as non-Hispanic white, non-Hispanic black, Mexican American, and others), smoking status (categorized as never, current, and former smoker), drinking status (classified into never drinker, current drinker (less than 20 g/day and 20 g or more/day), and former drinker according to alcohol intake), education background (classified into above high school, high school graduation/general educational development (GED), and less than high school), marital status (married or living with partner and living alone), hypertension status and diabetes status (obtained from self-report), energy intake, protein intake, carbohydrate intake, vitamin C intake, vitamin E intake, dietary fiber intake, BMI, serum creatinine, serum total cholesterol (STC), high-density lipoprotein cholesterol (HDL-C), glucose, and serum triglycerides (STG). 

### 2.3. Statistical Analyses

All statistical analyses were performed with the SAS version 9.4 (SAS Institute Inc., Cary, NC, USA). The continuous variable was expressed as median and Inter-Quartile Range, depending on the skewed distributed data. The categorical variable was presented as percentage. Wilcoxon signed-rank test was used to compare the zinc intake and the population RDAs. Differences between continuous variables were evaluated using the Wilcoxon rank sum test. Differences between categorical variables were assessed by the chi-square test and multiple comparisons based on Bonferroni correction. Multivariable logistic regression analysis was used to estimate the odds ratio(OR) and 95% confidence interval (CI) of HU according to the zinc intake quintile for male and female separately, Model 1 adjusted for age, race/ethnicity. Model 2 further adjusted for smoking status, drinking status, educational background, marital status, hypertension status, and diabetes status based on model 1. Model 3 further adjusted for energy intake, protein intake, carbohydrate intake, vitamin C intake, vitamin E intake, dietary fiber intake, STC, serum creatinine, glucose, BMI, HDL-C and STG based on model 2. A sensitivity analysis was undertaken using the second 24-h dietary recall data from 2003 to 2014 (*n* = 19,446). In this analysis, we used the mean of the nutrient intake from the two dietary recalls of and adjusted for the same covariates of the primary analyses. By and large, the relationships between the intake of zinc and HU were not altered. *p* value < 0.05 (two-sided) was regarded statistically significant, and *p* < 0.0125 (0.05/4), *p* < 0.0167 (0.05/3), *p* < 0.025 (0.05/2) was considered as statistical significance after Bonferroni adjustment for multiple comparisons.

## 3. Results

A total of 24,975 adult subjects were eventually enrolled in this study, which consisted of 12,757 males and 12,218 females. The daily zinc intake was 11.82 mg (8.12 mg–16.88 mg) for male, 8.45 mg (5.87 mg–11.87 mg) for females, all significantly higher than their respective RDAs (11.00 mg/day for male and 8.00 mg/day for females aged 19 years and above [26]) as is shown in Table 1. The characteristics of the participants according to those consuming less than RDAs of zinc and those consuming follow RDAs or greater for both sexes are shown in Table 2. For males, except for STC (*p* = 0.7974) and HDL-C (*p* = 0.1222), other indicators were all significantly different between two groups according to the intake of zinc. Compared to participants those consuming less than 11 mg zinc daily, those consuming 11 mg or greater daily were more likely to be younger, non-Hispanic white, above high school, current drinking (alcohol intake 20 g or more daily), heavier, taller, less likely to be non-Hispanic black, have less than high school education, live alone, be a former drinker, be a former smoker, less likely to have hypertension, diabetes and HU, have higher BMI, STG, and intakes of energy, protein, carbohydrate, vitamin C, vitamin E, dietary fiber, and have lower serum creatinine, glucose, and uric acid. For females, except for weight (*p* = 0.2418) and STG (*p* = 0.2392), other indicators were all significantly different between two groups according to the intake of zinc, compared to participants those consuming less than 8 mg zinc daily, those consuming 8 mg or greater daily were more likely to be younger, non-Hispanic white, have above high school education, be married or living with a partner, be a current drinker (alcohol intake less than 20 g/day and 20 g or more/day), less likely to be non-Hispanic black, high school or GED, less than high school, living alone, never drinking, former drinking, currently smoking, less likely to have hypertension, diabetes and HU, and have higher height, HDL-C, intakes of energy, protein, carbohydrate, vitamin C, vitamin E, dietary fiber, have lower BMI, STC, serum creatinine, glucose and uric acid. The results of comparing the nutrient intakes indicators between HU and non-HU are shown in Table 3. All of the nutrient intakes indicators were significantly different between HU and non-HU for male and female. Compared to the participants without HU, participants with HU had lower intakes of zinc, vitamin C, vitamin E, energy, protein, carbohydrate, and dietary fiber for both sexes.

Table 4 shows a significantly inverse association between zinc intake and HU was observed in the multivariable model. After controlling for age, race/ethnicity, smoking status, drinking status, education background, marital status, hypertension status and diabetes status, energy intake, protein intake, carbohydrate intake, vitamin C intake, vitamin E intake, dietary fiber intake, STC, serum creatinine, glucose, BMI, HDL-C, and STG. In males, compared with respondents consuming less than 7.33 mg zinc daily, the adjusted odds ratios (ORs) were 0.83 (95% CI, 0.71, 0.97) among those consuming 10.26–13.54 mg zinc daily, 0.78 (95% CI, 0.63–0.96) among those consuming 18.50 mg or greater, and *p* for the trend was 0.0134. For females, compared with respondents consuming less than 5.38 mg zinc daily, OR was 0.78 (95% CI,0.63, 0.97) among those consuming 9.64–12.93 mg zinc daily, and *p* for the trend was 0.3024.

The results of subgroup analysis are shown in Table 5. The inverse association between zinc intake and HU still existed in person without hypertension or diabetes. Adjusted ORs of HU among males without hypertension or diabetes in Q3 to Q5 of the zinc intake were 0.81 (95% CI, 0.67–0.97), 0.76 (95% CI, 0.62–0.93) and 0.73 (95% CI, 0.57–0.93), respectively, compared with Q1, and *P* for the trend was 0.0038. In females without hypertension or diabetes, the OR was 0.70 (95% CI, 0.56, 0.89) in Q2 compared with Q1 (*p* for trend = 0.2927). Nevertheless, no significant relationship between zinc intake and HU in both males and females with hypertension or diabetes. 

## 4. Discussion

In this large population-based study from the US adults, we observed a negative association between dietary zinc intake and HU in both men and women after adjustment for some potential confounders. Furthermore, the inverse associations between zinc intake and HU were observed in both males and females those without hypertension or diabetes, but no significant association was observed in males and females those suffering from hypertension or diabetes.

To the best of our knowledge, this is the first study to show an association between dietary zinc intake and HU in US population, and also the largest population-based study using a nationally representative sample. Several studies have indicated that low serum levels of zinc are associated with serum uric acid. Umeki et al. [18] reported that oral zinc therapy can normalize serum uric acid metabolism in Wilson’s disease through improving liver dysfunction and increasing uric acid synthesis. Besides, some animal studies reported that the serum uric acid levels of diabetic rats treated with zinc-flavonol complex were reverted back to near normalcy [19], and zinc supplementation or administration could normalize serum uric acid levels in rats with intestinal injury or aspirin-related damage [27,28]. In addition, Navarro-Alarcon et al. [20] reported that uric acid was negatively linearly related to serum zinc in hemodialysis patients in a longitudinal study. Although the relationship between serum zinc levels and risk of HU has been extensively studied, the evidence on the associations with dietary zinc intake is scarce. A cross-sectional study in China has shown a negative association between dietary zinc intake and HU in middle-aged and older males, but not in females [21], which is consistent with our findings that the negative association between HU and dietary zinc intake in man, but different from the inverse association was also observed in women in our result, major reasons for the inconsistent results can be explained due to difference of samples, difference of countries, and the age of the research participants also being different.

The underlying mechanisms of the association between dietary zinc intake and HU are largely unknown but maybe through the antioxidant properties. In biochemical systems, the antioxidant properties of zinc have been well demonstrated [17]. Nicotinamide adenine dinucleotide phosphate (NADPH) oxidases is inhibited by zinc, leading to reduced generation of reactive oxygen species (ROS), zinc is able to bind to sulfhydryl groups of various molecules, protecting them from oxidation [29]. Meanwhile, uric acid is a major antioxidant in the human plasma or pro-oxidant within the cell [16], and its concentration is almost 10-fold higher than other antioxidants. The major antioxidant effect of uric acid has been demonstrated is in the central nervous system, such as multiple sclerosis, Parkinson’s disease, and acute stroke [30,31,32,33], and the administration of uric acid decreased neutrophil infiltration and injury of the liver during hemorrhagic shock [34]. In addition, uric acid plays an important role in the pathogenesis of reactive oxygen species-related diseases [35], uric acid has been reported to stimulate increases nicotinamide adenine dinucleotide phosphate oxidase-derived reactive oxygen species production in adipocytes, vascular smooth muscle cells, as well as vascular endothelial cells [36,37]. Furthermore, uric acid may function as a pro-oxidant in HU, despite acting as an antioxidant under physiological conditions [38]. From the above analysis, it is speculated that dietary zinc intake maybe negatively associated with HU through the antioxidant properties. The underlying mechanisms of the association between dietary zinc intake and HU need to be clarified in future studies.

Previous studies showed the relationships between various food and HU. Inverse associations between intake of soy products [39], dairy products [40], and HU risk have been demonstrated. The consumption of vegetables and fruit—which are rich in dietary fiber, vitamin C, and folate—would effectively lower the risk of gout [41]. Nuts, legumes, and whole grains might be useful for protection against gout [42]. Our study showed that increased zinc intake may decrease the risks of HU. Zinc is present in a wide variety of foods [26], except seafood, red meat, and poultry, other food include whole grains, dairy products, baked beans, chickpeas, and nuts (such as cashews and almonds) are also good sources of zinc. Many ready-to-eat breakfast cereals are fortified with zinc [26]. In addition, vitamin C and vitamin E are also exogenous antioxidants, previous studies have demonstrated that vitamin C lowers serum uric acid level, higher vitamin C intake independently reduced gout risk in a prospective cohort study [43]. Previous observational studies have associated lower rates of heart disease with higher vitamin E intakes [44]. Vitamin E is a fat-soluble antioxidant that stops the production of ROS formed when fat undergoes oxidation [45]. In our study, participants with HU were had lower intakes of vitamin C and vitamin E than normal individuals.

Our results showed that the everyday intake of zinc in US adults was higher than the recommended amounts, and suggested the importance of RDAs for zinc. For males aged 19 years and above, the RDA for zinc is 11 mg/day, in our study, the adjusted OR was 0.83 among those consuming 10.26–13.54 mg zinc daily, compared with respondents consuming less than 7.33 mg zinc daily. For females aged 19 years and above, the RDA for zinc is 8 mg/day. In our results, the OR was 0.78 among those consuming 9.64–12.93 mg zinc daily, compared with respondents consuming less than 5.38 mg zinc daily. The reason may be that for pregnant and lactating women, the RDAs for zinc is higher than for other women (the RDAs for zinc is 11 mg/day for pregnant women and 12 mg/day for lactating women) because fetal requirements for high zinc and lactation can also deplete maternal zinc stores [46,47]. Pregnant women were excluded from our analysis, but lactating women which cannot be identified in our data were still in the analysis. Our findings suggested that adequate zinc intake may have a potential function for prevent or decrease the risk of HU.

Many studies have suggested that hypertension and diabetes may be an independent and crucial risk factor of HU [1,6]. Therefore, the present study analyzed the relationship between zinc intake and the risk of HU stratified by male and female participants with and without hypertension or diabetes. Consequently, only those without hypertension or diabetes showed an inverse association between dietary zinc intake and HU. This collection of evidence may provide an explanation for how these two diseases could attenuate the inverse association between dietary zinc intake and HU. The outcomes suggest that the existence of hypertension or diabetes may weaken the association between zinc intake and HU.

Our study has several strengths. First, to our knowledge, this is the first and largest nationally representative sample to assess the relationship between the zinc intake and HU in US adults. Second, our study adjusted for a wide range of potential confounding variables. Several limitations also need to be acknowledged. First, its cross-sectional design which was unable to determine causality or the temporal relationship between dietary zinc intake and HU. Second, although we adjusted for several major covariates in our analysis, the associations reported in our study may partially result from the potential confounding by other unobserved and unknown variables. Third, the 24-h dietary recall method was utilized to obtain dietary intake and may not reflect long-term zinc intake status, and diet misreporting—such as under-reporting and over-reporting—may occur. However, the similarity between the results in the sensitivity analysis using the mean of the two dietary recalls indicates that the effect of misclassification attributable to unmeasured variability was limited, and compared with food frequency questionnaires, 24-h recalls provide more food detail on the types and amounts. Fourth, the lack of plasma zinc and other zinc biomarker measures, however, blood levels may not entirely reveal nutritional status [48]. Fifth, our study was restricted to persons of European ancestry, and it is unknown whether our results can be generalized to other ethnic groups. Finally, further studies are needed to investigate the mechanism of this association.

## 5. Conclusions

The findings of this cross-sectional study indicated that dietary zinc intake is inversely associated with HU in US men and women, independent of some major confounding factors. In addition, this association remains valid for participants without hypertension or diabetes, but not for those with hypertension or diabetes.

## Figures and Tables

**Table 1:** Zinc intake among US adults (>19 years) in NHANES 2001–2014.

	RDAs for Zinc(mg/Day)	Zinc Intake(mg/Day)	*p*
Male (*n* = 12,757)	11.00	11.82 (8.12, 16.88)	<0.0001
Female (*n* = 12,218)	8.00	8.45 (5.87, 11.87)	<0.0001

**Table 2:** Characteristics of the participants according to intake of zinc.

Characteristic	Male		*p*	Female		*p*
	Zinc Intake <11 mg/Day (*n* = 5730)	Zinc Intake ≥11 mg/Day (*n* = 7027)		Zinc Intake <8 mg/Day (*n* = 5624)	Zinc Intake ≥8 mg/Day (*n* = 6594)	
Age (years)	51.00 (35.00, 65.00)	44.00 (32.00, 58.00)	<0.0001	50.00 (36.00, 64.00)	47.00 (33.00, 61.00)	<0.0001
Race/ethnicity (*n*, %)			<0.0001			<0.0001
Non-Hispanic white	2500 (43.63)	3712 (52.82)	<0.0001 ^c^	2571 (45.71)	3383 (51.30)	0.0003 ^c^
Non-Hispanic black	1291 (22.53)	1138 (16.19)	<0.0001 ^c^	1237 (22.00)	1130 (17.14)	<0.0001 ^c^
Mexican American	1011 (17.64)	1288 (18.33)	0.4037 ^c^	909 (16.16)	1137 (17.24)	0.1782 ^c^
Others ^a^	928 (16.20)	889 (12.65)	<0.0001 ^c^	907 (16.13)	944 (14.32)	0.0170 ^c^
Education background (*n*, %)			<0.0001			<0.0001
Above high School	2656 (46.35)	3676 (52.31)	<0.0001 ^d^	2737 (48.67)	3769 (57.16)	<0.0001 ^d^
High school or GED ^b^	1344 (23.46)	1722 (24.51)	0.2797 ^d^	1341 (23.84)	1411 (21.40)	0.0104 ^d^
Less than high School	1730 (30.19)	1629 (23.18)	<0.0001 ^d^	1546 (27.49)	1414 (21.44)	<0.0001 ^d^
Marital status (*n*, %)			0.0001			<0.0001
Married or living with partner	3703 (64.62)	4767 (67.84)	0.0855 ^e^	3001 (53.36)	3786 (57.42)	0.0161 ^e^
Living alone	2027 (35.38)	2260 (32.16)	0.0071 ^e^	2623 (46.64)	2808 (42.58)	0.0053 ^e^
Drinking status (*n*, %)			<0.0001			<0.0001
Never	431 (7.52)	477 (6.79)	0.1356 ^c^	1203 (21.39)	1144 (17.35)	<0.0001 ^c^
Current (<20 g/day)	3529 (61.59)	4245 (60.41)	0.5040 ^c^	2771 (49.27)	3520 (53.38)	0.0102 ^c^
Current (≥20 g/day)	1207 (21.06)	1755 (24.98)	<0.0001 ^c^	501 (8.91)	749 (11.36)	<0.0001 ^c^
Former	563 (9.83)	550 (7.83)	0.0003 ^c^	1149 (20.43)	1181 (17.91)	0.0036 ^c^
Smoking status (*n*, %)			0.0008			0.0006
Never	2482 (43.32)	3241 (46.12)	0.0503 ^d^	3421 (60.83)	4104 (62.24)	0.4361 ^d^
Current	1505 (26.27)	1847 (26.28)	0.9852 ^d^	1148 (20.41)	1170 (17.74)	0.0020 ^d^
Former	1743 (30.42)	1939 (27.59)	0.0093 ^d^	1055 (18.76)	1320 (20.02)	0.1497 ^d^
Weight (kg)	82.40 (72.10, 94.60)	84.80 (74.40, 97.30)	<0.0001	71.40 (61.10, 85.00)	71.60 (61.30, 85.30)	0.2418
Height (cm)	173.60 (168.60, 178.80)	175.50 (170.40, 180.70)	<0.0001	160.50 (155.50, 165.10)	161.70 (156.70, 166.40)	<0.0001
BMI (kg/m^2^)	27.40 (24.40, 30.80)	27.70 (24.51, 31.14)	0.0114	27.90 (23.95, 32.81)	27.46 (23.60, 32.58)	0.0080
Hypertension status (*n*, %)	1412 (24.64)	1322 (18.81)	<0.0001	1307 (23.24)	1229 (18.64)	<0.0001
Diabetes status (*n*, %)	608 (10.61)	577 (8.21)	<0.0001	609 (10.83)	561 (8.51)	<0.0001
HU (*n*, %)	1309 (22.84)	1383 (19.68)	<0.0001	986 (17.53)	959 (14.54)	<0.0001
Energy intake (kcal/day)	1792.00 (1389.00, 2268.00)	2824.00 (2261.00, 3554.00)	<0.0001	1340.00 (1033.00, 1681.00)	2036.00 (1643.00, 2524.00)	<0.0001
Protein intake (g/day)	63.44 (47.88, 80.69)	112.73 (90.27, 141.69)	<0.0001	46.51 (35.22, 58.28)	79.91 (64.77, 98.90)	<0.0001
Carbohydrate intake (g/day)	223.34 (164.40, 292.01)	325.89 (246.37, 426.19)	<0.0001	172.31 (127.39, 224.84)	245.76 (189.64, 313.61)	<0.0001
Vitamin C intake (mg/day)	44.00 (16.60, 109.40)	68.70 (30.20, 146.80)	<0.0001	39.10 (15.10, 93.75)	64.65 (29.10, 128.00)	<0.0001
Vitamin E intake (mg/day)	5.22 (3.40, 7.89)	8.63 (5.81, 12.64)	<0.0001	4.32 (2.79, 6.42)	6.98 (4.73, 10.15)	<0.0001
Dietary fiber intake (g/day)	12.20 (8.00, 17.70)	19.60 (13.60, 27.60)	<0.0001	10.10 (6.70, 14.30)	16.10 (11.40, 22.40)	<0.0001
STC (mg/dL)	192.00 (165.00, 220.00)	192.00 (166.00, 220.00)	0.7974	197.00 (171.00, 226.00)	196.00 (171.00, 223.00)	0.0298
Serum creatinine (mg/dL)	0.99 (0.87, 1.10)	0.97 (0.86, 1.09)	<0.0001	0.76 (0.67, 0.87)	0.74 (0.66, 0.83)	<0.0001
Glucose (mg/dL)	94.00 (87.00, 104.00)	93.00 (86.00, 103.00)	<0.0001	91.00 (84.00, 101.00)	90.00 (83.00, 100.00)	0.0011
STG (mg/dL)	125.00 (82.00, 194.00)	128.00 (84.00, 204.00)	0.0008	111.00 (75.00, 165.00)	108.00 (74.00, 166.00)	0.2392
HDL-C (mg/dL)	46.00 (39.00, 55.00)	46.00 (39.00, 55.00)	0.1222	55.00 (46.00, 67.00)	56.00 (46.00, 67.00)	0.0201
Uric acid (mg/dL)	6.00 (5.20, 6.90)	5.90 (5.20, 6.70)	<0.0001	4.70 (4.00, 5.60)	4.60 (3.90, 5.40)	<0.0001

^a^ Other Hispanics and other races including multi-racial participants; ^b^ General Educational Development; ^c^ Statistically significant after Bonferonni adjustment (0.05/4 = 0.0125); ^d^ Statistically significant after Bonferonni adjustment (0.05/3 = 0.0167); ^e^ Statistically significant after Bonferonni adjustment (0.05/2 = 0.025).

**Table 3:** Nutrient intakes characteristics of participants with or without HU.

Characteristic	Male		*p*	Female		*p*
	Non-HU (*n* = 10,065)	HU (*n* = 2692)		Non-HU (*n* = 10,273)	HU (*n* = 1945)	
Zinc intake (mg/day)	11.95 (8.22, 17.12)	11.26 (7.69,16.23)	<0.0001	8.55 (5.96, 11.96)	7.93 (5.44, 11.31)	<0.0001
Vitamin C intake (mg/day)	59.90 (23.90, 132.90)	50.15 (20.40, 116.80)	<0.0001	54.40 (22.00, 115.00)	46.80 (19.00, 98.60)	<0.0001
Vitamin E intake (mg/day)	7.08 (4.55, 10.75)	6.45 (4.10, 9.89)	<0.0001	5.72 (3.68, 8.57)	5.22 (3.30, 7.89)	<0.0001
Energy intake (kcal/day)	2356.00 (1749.00, 3075.00)	2244.50 (1686.50, 2971.50)	<0.0001	1709.00 (1300.00, 2209.00)	1579.00 (1201.00, 2068.00)	<0.0001
Carbohydrate intake (g/day)	279.96 (205.22, 375.88)	257.69 (183.89, 349.50)	<0.0001	213.61 (157.48, 279.49)	191.97 (140.12, 253.18)	<0.0001
Protein intake (g/day)	88.85 (64.44, 119.84)	86.01 (61.26, 116.79)	0.0015	63.63 (46.54, 84.77)	60.63 (43.75, 80.15)	<0.0001
Dietary fiber intake (g/day)	16.40 (10.80, 23.90)	14.00 (9.00, 21.20)	<0.0001	13.30 (8.80, 19.10)	11.90 (8.00, 17.00)	<0.0001

**Table 4:** Adjusted odds ratios of HU among participants associated with zinc intake

		Zinc Intake(mg/Day)					*p* for Trend
		Q1 (≤7.33)	Q2 (7.34–10.25)	Q3 (10.26–13.54)	Q4 (13.55–18.49)	Q5 (≥18.50)	
		(*n* = 2553)	(*n* = 2551)	(*n* = 2554)	(*n* = 2551)	(*n* = 2548)	
Male (*n* = 12,757)	Model 1	Reference	0.95 (0.83,1.08)	0.85 (0.74,0.97)	0.82 (0.71,0.94)	0.76 (0.66, 0.87)	<0.0001
	Model 2	Reference	0.93 (0.81,1.06)	0.82 (0.72, 0.94)	0.80 (0.69,0.91)	0.74 (0.65, 0.86)	<0.0001
	Model 3	Reference	0.93 (0.81,1.07)	0.83 (0.71, 0.97)	0.85 (0.71, 1.00)	0.78 (0.63, 0.96)	0.0134
		Q1 (≤5.38)	Q2 (5.39–7.37)	Q3 (7.38–9.63)	Q4 (9.64–12.93)	Q5 (≥12.94)	
		(*n* = 2445)	(*n* = 2444)	(*n* = 2451)	(*n* = 2437)	(*n* = 2441)	
Female (*n* = 12,218)	Model 1	Reference	0.81(0.70, 0.94)	0.90(0.77, 1.04)	0.73(0.62, 0.86)	0.85(0.72, 0.99)	0.0119
	Model 2	Reference	0.83(0.71, 0.97)	0.91(0.79, 1.07)	0.75(0.64, 0.88)	0.86(0.74, 1.01)	0.0255
	Model 3	Reference	0.90(0.76, 1.08)	0.98(0.82, 1.18)	0.78(0.63, 0.97)	0.94(0.74, 1.19)	0.3024

Q1–Q5: quintiles 1 to 5; Model 1 adjusted for age, race/ethnicity; Model 2 adjusted for age, race/ethnicity, smoking status, drinking status, education background, marital status, hypertension status and diabetes status; Model 3 adjusted for age, race/ethnicity, smoking status, drinking status, education background, marital status, hypertension status and diabetes status, energy intake, protein intake, carbohydrate intake, vitamin C intake, vitamin E intake, dietary fiber intake, STC, serum creatinine, glucose, BMI, HDL-C, and STG.

**Table 5:** Subgroup analysis by stratifying the data of participants with or without hypertension or diabetes

		Zinc Intake(mg/Day)					*p* for Trend
Male (*n* = 12,757)	*n* = 9197	Q1 (≤7.67)	Q2 (7.68–10.66)	Q3 (10.67–13.98)	Q4 (13.99–19.08)	Q5 (≥19.09)	
		(*n* = 1840)	(*n* = 1844)	(*n* = 1839)	(*n* = 1838)	(*n* = 1836)	
	Model 4 ^a^	Reference	0.91 (0.76, 1.08)	0.81 (0.67, 0.97)	0.76 (0.62, 0.93)	0.73 (0.57, 0.93)	0.0038
	*n* = 3560	Q1 (≤6.60)	Q2 (6.61–9.46)	Q3 (9.47–12.42)	Q4 (12.43–16.99)	Q5 (≥17.00)	
		(*n* = 713)	(*n* = 712)	(*n* = 711)	(*n* = 712)	(*n* = 712)	
	Model 4 ^b^	Reference	0.99 (0.76, 1.29)	0.82 (0.61, 1.09)	0.87 (0.64, 1.19)	0.96 (0.66, 1.39)	0.5293
Female (*n* = 12,218)	*n* = 8910	Q1 (≤5.54)	Q2 (5.55–7.54)	Q3 (7.55–9.86)	Q4 (9.87–13.17)	Q5 (≥13.18)	
		(*n* = 1784)	(*n* = 1786)	(*n* = 1779)	(*n* = 1781)	(*n* = 1780)	
	Model 4 ^a^	Reference	0.70 (0.56, 0.89)	1.04 (0.83, 1.32)	0.78 (0.60, 1.01)	0.77 (0.56, 1.04)	0.2927
	*n* = 3308	Q1 (≤5.01)	Q2 (5.02–6.98)	Q3 (6.99–9.03)	Q4 (9.04–12.20)	Q5 (≥12.21)	
		(*n* = 667)	(*n* = 657)	(*n* = 662)	(*n* = 661)	(*n* = 661)	
	Model 4 ^b^	Reference	1.01 (0.76, 1.34)	0.99 (0.72, 1.35)	0.72 (0.51, 1.01)	1.04 (0.70, 1.54)	0.4467

Q1–Q5: quintiles 1 to 5; Model 4 adjusted for age, race/ethnicity, smoking status, drinking status, education background, marital status, energy intake, protein intake, carbohydrate intake, vitamin C intake, vitamin E intake, dietary fiber intake, STC, serum creatinine, glucose, BMI, HDL-C and STG. ^a^ participants without hypertension or diabetes; ^b^ participants with hypertension or diabetes.
